# Gaussian quantum states can be disentangled using symplectic rotations

**DOI:** 10.1007/s11005-021-01410-4

**Published:** 2021-05-24

**Authors:** Maurice A. de Gosson

**Affiliations:** Faculty of Mathematics (NuHAG), University of Vienna, Vienna, Austria

**Keywords:** Entanglement, Disentanglement, Gaussian state, Density operator, Symplectic rotation, 81Qxx, 91S10, 46N50

## Abstract

We show that every Gaussian mixed quantum state can be disentangled by conjugation with a passive symplectic transformation, that is a metaplectic operator associated with a symplectic rotation. The main tools we use are the Werner–Wolf condition on covariance matrices and the symplectic covariance of Weyl quantization. Our result therefore complements a recent study by Lami, Serafini, and Adesso.

## Introduction

Gaussian states are important for quantum information processing. Advances in the study of their entanglement properties is useful and of interest to the community working in the area of continuous variable systems which typically get realized in quantum optics. While it is well-known that such states can be made separable (“disentangled”) by diagonalizing the covariance matrix by a symplectic transformation (Williamson normal form), we prove a much stronger result, namely that this disentanglement can.be made by using a symplectic rotation (or “passive symplectic transformation”, to use the physicists’ jargon).

The main tools we use are a diagonalization result for positive definite symplectic matrices (Lemma 2) and the symplectic covariance of the Wigner transform. We begin by proving the result for bipartite states (Theorem 4), the multipartite readily follows (Corollary 6 ). Theorem 4 has been announced in a *Note aux Comptes Rendus de l’Académie des Sciences de Paris *[12].

### Notation

Let $\mathbb {R}^{2n}=\mathbb {R}^{2n_{A}}\oplus \mathbb {R}^{2n_{B}}$ be the phase space of a bipartite system ($n_{A}\ge 1$, $n_{B}\ge 1$). We will use the following phase space variable ordering: $z=(z_{A},z_{B})=z_{A}\oplus z_{B}$ with $z_{A}=(x_{1},p_{1},\ldots ,x_{n_{A}},p_{n_{A}})$ and $z_{B} =(x_{n_{A}+1},p_{n_{A}+1},\ldots ,x_{n},p_{n})$. We equip the symplectic spaces $\mathbb {R}^{2n_{A}}$ and $\mathbb {R}^{2n_{B}}$ with their canonical bases. The symplectic form on $\mathbb {R}^{2n}$ is

$$\begin{aligned} \sigma =\mathrm{{d}}p_{1}\wedge \mathrm{{d}}x_{1}+\cdots +\mathrm{{d}}p_{n}\wedge \mathrm{{d}}x_{n}, \end{aligned}$$

equivalently, $\sigma (z,z^{\prime })=Jz\cdot z^{\prime }$, and the corresponding symplectic group is denoted : $S\in {\text {*}}{Sp}(2n,\mathbb {R})$ if and only if $S\in \mathrm{{GL}}(2n,\mathbb {R} )$. In the splitting $\mathbb {R}^{2n}=\mathbb {R}^{2n_{A}}\oplus \mathbb {R} ^{2n_{B}}$ we have $J=J_{A}\oplus J_{B}$ where

$$\begin{aligned} J_{A}=\bigoplus _{k=1}^{n_{A}}J_{k}\text { \ },\text {\ }J_{k}= \begin{pmatrix} 0 &{}\quad 1\\ -1 &{}\quad 0 \end{pmatrix} \end{aligned}$$

and likewise for $J_{B}$. Thus $J_{A}$ and $J_{B}$ determines the symplectic structures $\sigma _{A}$ and $\sigma _{B}$ on the partial phase spaces $\mathbb {R}^{2n_{A}}$ and $\mathbb {R}^{2n_{B}}$.

## Separability and entanglement

The study of entanglement and separability of continuous-variable systems is at the heart of quantum information theory and of quantum optics [1]. Of particular interest are Gaussian states [2, 3]. We gather here the essentials from the theory of mixed quantum states we will need in the rest of the paper. Excellent texts about the topic abound in the literature, see for instance [1, 2, 11, 14, 16]. Quantum states are identified with their density operators $\widehat{\rho }$. A density operator is a positive semidefinite and self-adjoint operator with unit trace on a Hilbert space $\mathcal {H}$; it is hence a compact operators. Every density operator $\widehat{\rho }$ can be written (non-uniquely) as a convex sum

1 $$\begin{aligned} \widehat{\rho }=\sum _{j}\lambda _{j}|\psi _{j}\rangle ~\langle \psi _{j}|=\sum _{j}\lambda _{j}\Pi _{j} \end{aligned}$$

where $\Pi _{j}=|\psi _{j}\rangle ~\langle \psi _{j}|$ is the orthogonal projection on the ray $\mathbb {C}\psi _{j}$ (we assume the $\psi _{j}\in \mathcal {H}$ are normalized vectors). When $\mathcal {H}=L^{2}(\mathbb {R}^{n})$, which we assume from now on, the Wigner distribution of $\widehat{\rho }$ is the function $W_{\widehat{\rho }}\in L^{2}(\mathbb {R}^{2n})$ defined by

2 $$\begin{aligned} W_{\widehat{\rho }}(z)=\left( \tfrac{1}{2\pi \hbar }\right) ^{n}\int \mathrm{{e}}^{-\frac{i}{\hbar }py}\langle x+\tfrac{1}{2}y|\widehat{\rho }|x-\tfrac{1}{2}y\rangle \mathrm{{d}}^{n}y\, \end{aligned}$$

($$z=(x,p)$$), that is

3 $$\begin{aligned} W_{\widehat{\rho }}=\sum _{j}\lambda _{j}W\psi _{j} \end{aligned}$$

(we assume $W\psi _{j}\in L^{1}(\mathbb {R}^{n})\cap L^{2}(\mathbb {R}^{n})$) where $W\psi _{j}$ is the usual Wigner transform of $\psi _{j}$:

4 $$\begin{aligned} W\psi _{j}(z)=\left( \tfrac{1}{2\pi \hbar }\right) ^{n}\int \mathrm{{e}}^{-\frac{i}{\hbar }py}\psi _{j}(x+\tfrac{1}{2}y)\psi _{j}^{*}(x-\tfrac{1}{2}y)\mathrm{{d}}^{n}y. \end{aligned}$$

A quantum state consisting of two parts *A* and *B* is called separable (with respect to the partition *AB*) if there exist sequences of density operators $(\widehat{\rho }_{j}^{A})$ and $(\widehat{\rho }_{j}^{B})$ on $L^{2} (\mathbb {R}^{n_{A}})$ and $L^{2}(\mathbb {R}^{n_{B}})$, respectively, and coefficients $\lambda _{j}^{AB}\ge 0$ summing up to one, such that

5 $$\begin{aligned} \widehat{\rho }=\sum _{j}\lambda _{j}^{AB}\widehat{\rho }_{\ell j}^{A} \otimes \widehat{\rho }_{j}^{B} \end{aligned}$$

where the convergence is in the trace-class norm. When $\widehat{\rho }$ is not separable, it is called an *entangled state*. The problem of determining necessary and sufficient conditions for a density operator to be separable is still very largely open; while there exist necessary conditions (in particular the so-called PPT condition [1]), no simple sufficient condition for separability is known in the general case; for a recent up to date discussion see Lami *et al*. [18].

## The covariance matrix of a Gaussian bipartite state

Let $\Sigma$ be a real positive definite symmetric $2n\times 2n$ matrix (to be called “covariance matrix” from now on) and consider the associated normal probability distribution centered at $\mu$:

6 $$\begin{aligned} W_{\widehat{\rho }}(z)=\frac{1}{(2\pi )^{n}\sqrt{\det \Sigma }}\mathrm{{e}}^{-\frac{1}{2}\Sigma ^{-1}(z-\mu )^{2}}. \end{aligned}$$

If the covariance matrix satisfies in addition the condition

7 $$\begin{aligned} \Sigma +\frac{i\hbar }{2}J\ge 0 \end{aligned}$$

($A\ge B$ meaning that $A-B$ is positive semidefinite) then $W_{\widehat{\rho }}$ is the Wigner distribution of a mixed quantum state, identified with its density operator $\widehat{\rho }$ via (1) and (2). We notice, in passing, that property (7), which ensures that the associated density operator $\widehat{\rho }$ is positive semidefinite, crucially depends on the numerical value of $\hbar$ (see [10, 13]). When $\widehat{\rho }$ is a Gaussian state one shows [1, 18, 21] that a necessary and sufficient condition for bipartite *AB*-separability is the existence of a $2n_{A}\times 2n_{A}$ covariance matrix $\Sigma _{A}$ and a $2n_{B}\times 2n_{B}$ covariance matrix $\Sigma _{B}$ such that

8 $$\begin{aligned}&\Sigma _{A}+\frac{i\hbar }{2}J_{A}\ge 0\text { } \end{aligned}$$

9 $$\begin{aligned}&\text {\ }\Sigma _{B}+\frac{i\hbar }{2}J_{B}\ge 0\text {\ } \end{aligned}$$

10 $$\begin{aligned}&\Sigma \ge \Sigma _{A}\oplus \Sigma _{B}. \end{aligned}$$

Testing numerically these conditions can be viewed as problem in convex optimization: see for instance Giedke et al. [16] or Hyllus and Eisert [17]. Lami et al. [18] have simplified the condition (10) by showing that it can be replaced with the simpler relation $\Sigma \ge \Sigma _{A}\oplus \frac{i\hbar }{2}J_{B}$ which considerably reduces its computational complexity (testing for separability in the general case is known to be a NP-hard problem).

## Symplectic rotations

Among all linear symplectic transformations of a quantum state, a special role is played by those which in addition orthogonal. In addition to their genuine theoretical importance, their practical interest comes from the fact that they can be experimentally implemented using only beam splitters and phase plates [14, 18]. In Lami et al. [18] have pursued a study initiated by Wolf et al. [22] who asked which Gaussian states can be entangled by passive transformations. In particular, Lami et al. characterize the Gaussian states that are separable for all symplectic rotations. To prove our main result we need some facts from symplectic geometry; for details see e.g., [5, 8].

### The subgroup *U*(*n*)

The natural embedding $M(n,\mathbb {C})\longrightarrow M(2n,\mathbb {R})$ given by

$$\begin{aligned} A+iB\longmapsto \begin{pmatrix} A &{}\quad -B\\ B &{}\quad A \end{pmatrix} \end{aligned}$$

identifies the unitary group $U(n,\mathbb {C})$ with a (maximally compact) subgroup *U*(*n*) of ; its elements will be called *symplectic rotations*. This terminology is justified by the fact that one has [8]in the quantum mechanical literature the elements of *U*(*n*) are also called *passive symplectic transformations*. As the relation $S^{T}JS=J$ characterizes the elements of , we have $R\in U(n)$ if and only  and $RJ=JR$.

The two following well-known [5, 8, 15] elementary results are essential for the proof of Theorem 4:

#### Lemma 1

(Polar decomposition) Every  can be written uniquely as a product $S=PR$ where  is positive definite ($P>0$) and $R\in U(n)$. The automorphisms *P* and *R* are explicitly given by

11 $$\begin{aligned} P=\big (S^{T}S\big )^{1/2}\text { \ },\text {\ }R=\big (S^{T}S\big )^{-1/2}S. \end{aligned}$$

#### Proof

It is clear that $S=PR$ and that  and $P>0$. Let us show that $R\in U(n)$. Since the unique square root of a positive symplectic matrix is also symplectic [8] we have  and it is thus sufficient to show that $RR^{T}=I$. But this follows from the obvious equality

$$\begin{aligned} RR^{T}=\big (S^{T}S\big )^{-1/2}SS^{T}\big (S^{T}S\big )^{-1/2}=I. \end{aligned}$$

The uniqueness of the decomposition $S=PR$ follows from the uniqueness of general polar decompositions. $\square$

#### Lemma 2

(Diagonalization) Let  be positive definite. (i) The eigenvalues of *P* are all positive numbers; If $\lambda$ is an eigenvalue of *P*, then so is $1/\lambda$; (ii) Let $0<\lambda _{1}\le \lambda _{2}\le \cdots \le \lambda _{n}$ be the *n* smallest eigenvalues of *P*. There exists $U\in U(n)$ such that

12 $$\begin{aligned} P=U^{T}\Delta U \end{aligned}$$

where *D* is the diagonal matrix13

#### Proof

See [8], Sect. 2.1, Prop. 2.13, where an explicit construction of the diagonalizing matrix *U* is given. The fact that the eigenvalues come in pairs $(\lambda _{j},\lambda _{j}^{-1})$ is due to the fact that the characteristic polynomial of a symplectic matrix is reflexive; that the $\lambda _{j}$ are positive follows from $P>0$ [4, 8, 15]. $\square$

Formula (12) is reminiscent of the Bloch–Messiah (or Euler) decomposition [7] used in the reduction of unitaries [6]. The latter says that every  can be written as $S=UDU^{\prime }$ with $U\in U(n)$ and *D* diagonal; our result, which applies to positive definite symplectic matrices is much stronger since it implies $U^{\prime }=U$ and gives an explicit construction of the diagonal matrix.

### The metaplectic representation of 

The symplectic group  is a classical connected Lie group having coverings of all orders (this follows from the fact that  is contractible to its maximal compact group, *U*(*n*), which implies that  and *U*(*n*) have isomorphic first homotopy groups [8]). It turns out that the double covering  has a faithful representation by a (connected) group of unitary operators on $L^{2} (\mathbb {R}^{n})$, the metaplectic group . There are several ways to describe . The simplest [8, 15] is to note that a set of generators of  consists of the symplectic matrices

$$\begin{aligned} V_{-P}&= \begin{pmatrix} I &{}\quad 0\\ P &{}\quad I \end{pmatrix} { \ , \ }P=P^{T}\\ M_{L}&= \begin{pmatrix} L^{-1} &{}\quad 0\\ 0 &{}\quad L^{T} \end{pmatrix} { \ , \ }\det L\ne 0 \end{aligned}$$

together with the standard symplectic matrix *J*; the corresponding metaplectic operators (which also generate ) are then the unitary operators $\pm \widehat{V}_{-P}$, $\pm \widehat{M} _{L,m}$, and $\pm \widehat{J}$ where

$$\begin{aligned} \widehat{V}_{-P}\psi (x)= & {} \mathrm{{e}}^{-iPx^{2}/2\hbar }\psi (x)\\ \widehat{M}_{L,m}\psi (x)= & {} i^{m}\sqrt{|\det L|}\psi (x)\\ \widehat{J}\psi (x)= & {} \left( \tfrac{1}{2\pi i\hbar }\right) ^{n/2}\int \mathrm{{e}}^{-ixx^{\prime }/\hbar }\psi (x^{\prime })\mathrm{{d}}^{n}x^{\prime } \end{aligned}$$

where the integer *m* in the second equation is defined by $m\pi =\arg \det L$.

Recall now the following symplectic covariance property (see for instance [19] or [8], Ch.7). Let $\widehat{A}=\mathrm {Op} ^{\mathrm {W}}(a)$ be the Weyl quantization of an observable *a*; formally:

$$\begin{aligned} \widehat{A}\psi (x)=\left( \tfrac{1}{2\pi \hbar }\right) ^{n}\iint \mathrm{{e}}^{-ipy/\hbar }a\left( \frac{1}{2}(x+y),p\right) \psi (y)\mathrm{{d}}^{n}p\mathrm{{d}}^{n}y. \end{aligned}$$

For every  with projection  we have

14 $$\begin{aligned} \widehat{S}\mathrm {Op}^{\mathrm {W}}(a)\widehat{S}^{-1}=\mathrm {Op} ^{\mathrm {W}}\left( a\circ S^{-1}\right) . \end{aligned}$$

A density operator $\widehat{\rho }$ being the quantization up to the factor $(2\pi \hbar )^{n}$ of its Wigner distribution $W_{\widehat{\rho }}$:

15 $$\begin{aligned} \widehat{\rho }=(2\pi \hbar )^{n}\mathrm {Op}^{\mathrm {W}}(W_{\widehat{\rho }}) \end{aligned}$$

formula (14) reads

16 $$\begin{aligned} \widehat{S}\widehat{\rho }\widehat{S}^{-1}=\widehat{W_{\widehat{\rho }}\circ S^{-1}}. \end{aligned}$$

### A simple example

The example which follows is in a sense trivial, and the result is probably well-known. It however contains the main ideas which will lead to a proof of the main result (Theorem 4). We consider a (centered) Gaussian state $|\psi _{X,Y}\rangle$ where

17 $$\begin{aligned} \psi _{X,Y}(x)=\left( \tfrac{1}{\pi \hbar }\right) ^{n/4}(\det X)^{1/4} \mathrm{{e}}^{-\tfrac{1}{2\hbar }(X+iY)x^{2}}; \end{aligned}$$

here *X* and *Y* are real symmetric $n\times n$ matrices with $X>0$. Its Wigner transform is well-known [8, 19], it is the phase space Gaussian

18 $$\begin{aligned} W\psi _{X,Y}(z)=\left( \tfrac{1}{\pi \hbar }\right) ^{n}\mathrm{{e}}^{-\tfrac{1}{\hbar }S^{T}Sz^{2}} \end{aligned}$$

where  is given by

19 $$\begin{aligned} S= \begin{pmatrix} X^{1/2} &{}\quad 0\\ X^{-1/2}Y &{}\quad X^{-1/2} \end{pmatrix} . \end{aligned}$$

Setting $P=S^{T}S$ we know that in view of Lemma 1 there exists $U\in U(n)$ such that $P=U^{T}\Delta U$ where $\Delta$ has the diagonal form (13). We can thus rewrite (18) as

20 $$\begin{aligned} W(\psi _{X,Y})(U^{-1}z)=W(\widehat{U}\psi _{X,Y})(z)=\left( \tfrac{1}{\pi \hbar }\right) ^{n}\mathrm{{e}}^{-\tfrac{1}{\hbar }\Delta z^{2}} \end{aligned}$$

where we have taken into account the symplectic covariance properties of the Wigner transform [8, 19]. Thus,

$$\begin{aligned} \widehat{U}\psi _{X,Y}=\psi _{1}\otimes \psi _{2}\otimes \cdots \otimes \psi _{n} \end{aligned}$$

where the $\psi _{j}$ are the squeezed states

$$\begin{aligned} \psi _{j}(x_{j})=(\pi \hbar )^{-1/4}\mathrm{{e}}^{-\lambda _{j}x_{j}^{2}/\hbar }. \end{aligned}$$

## The main results

We begin by studying the bipartite case. We thereafter extend it to multipartite Gaussian states where the notation is somewhat more cumbersome.

### Statement and proof

We are going to prove that for every Gaussian bipartite state there exists a metaplectic operator $\widehat{U}$ associated with a symplectic rotation *U* and such that $\widehat{U}\widehat{\rho }\widehat{U}^{-1}$ is a separable Gaussian state. We begin by recalling the following geometric result [8, 9]:

#### Lemma 3

The quantum condition (7) is equivalent to the statement:21where $B^{2n}(\sqrt{\hbar })$ is the phase space ball defined by $|z|^{2} \le \hbar$ and $\Omega _{\Sigma }$ is the covariance ellipsoid of $\widehat{\rho }$:

$$\begin{aligned} \Omega _{\Sigma }=\{z\in \mathbb {R}^{2n}:\tfrac{1}{2}\Sigma ^{-1}z^{2}\le 1\}. \end{aligned}$$

The proof of property (21) makes use of the properties of the symplectic spectrum of the covariance matrix.

#### Theorem 4

Let $\widehat{\rho }$ be a bipartite Gaussian density operator. There exists a symplectic rotation $U\in U(n)$ such that $\widehat{U} \widehat{\rho }\widehat{U}^{-1}$ is separable where  is any one of the two metaplectic operators covering *U*.

#### Proof

Let $S=PR$ be the symplectic polar decomposition (Lemma 1) of , that is , $P>0$, and $R\in U(n)$. We have $SB^{2n}(\sqrt{\hbar })=PB^{2n}(\sqrt{\hbar })$ by rotational symmetry of the ball $B^{2n} (\sqrt{\hbar })$. In view of Lemma 2 there exists a symplectic rotation $U\in U(n)$ diagonalizing *P*:

22 $$\begin{aligned} P=U^{T}\Delta U \end{aligned}$$

where  is the diagonal matrix (13) whose form will be discussed in a moment. The inclusion $SB^{2n}(\sqrt{\hbar })\subset \Omega _{\Sigma }$ in (21) is thus equivalent to $\Delta B^{2n}(\sqrt{\hbar })\subset U(\Omega _{\Sigma })$, that is to

23 $$\begin{aligned} \Delta B^{2n}(\sqrt{\hbar })\subset \Omega _{\Sigma _{U}} \end{aligned}$$

where $\Sigma _{U}=U\Sigma U^{T}$. This inclusion is equivalent to the matrix inequality

24 $$\begin{aligned} \frac{\hbar }{2}\Delta ^{2}\le \Sigma _{U}. \end{aligned}$$

We next note that $\Sigma _{U}$ is the covariance matrix of the density operator $\widehat{\rho }_{U}$ with Wigner distribution $W_{\widehat{\rho } U}(z)=W_{\widehat{\rho }}(U^{T}z)$ that is

25 $$\begin{aligned} W_{\widehat{\rho }U}(z)=\frac{1}{(2\pi )^{n}\sqrt{\det U\Sigma U^{T}}} \mathrm{{e}}^{-\frac{1}{2}\Sigma ^{-1}U^{T}z\cdot U^{T}z}. \end{aligned}$$

Applying the symplectic covariance formula (16) for Weyl operators to $\widehat{\rho }$ yields, since $U^{T}=U^{-1}$,

26 $$\begin{aligned} \widehat{\rho }_{U}=\widehat{U}\widehat{\rho }\widehat{U}^{-1} \end{aligned}$$

where  is anyone of the two metaplectic operators $\pm \widehat{U}$ covering *U*. We claim that $\widehat{\rho }_{U}$ is separable. To see this, let us come back to the diagonal matrix $\Delta$ appearing in the factorization (22). It is given by27and in the *AB*-ordering, it has the form $\Delta =\Delta _{A}\oplus \Delta _{B}$ with

28 $$\begin{aligned} \Delta _{A}=\bigoplus _{k=1}^{n_{A}}\Delta _{k}{ \ , \ }\Delta _{B} =\bigoplus _{k=n_{A}+1}^{n}\Delta _{k} \end{aligned}$$

and

29 $$\begin{aligned} \Delta _{k}= \begin{pmatrix} \lambda _{k} &{}\quad 0\\ 0 &{}\quad \lambda _{k}^{-1} \end{pmatrix} { \ , \ }k=1,\ldots ,n. \end{aligned}$$

Clearly  and . The symmetric matrices

30 $$\begin{aligned} \Sigma _{A}=\frac{\hbar }{2}\Delta _{A}^{2}\text { \ },\text { \ }\Sigma _{B} =\frac{\hbar }{2}\Delta _{B}^{2} \end{aligned}$$

trivially satisfy

31 $$\begin{aligned} \Sigma _{A}+\frac{i\hbar }{2}J_{A}\ge 0{ \ , \ }\Sigma _{B}+\frac{i\hbar }{2}J_{B}\ge 0. \end{aligned}$$

In view of (24), we have

32 $$\begin{aligned} \Sigma _{A}\oplus \Sigma _{B}\le \Sigma _{U} \end{aligned}$$

and the theorem now follows using the separability conditions (8)–(10). $\square$

#### Remark 5

The eigenvalues $\lambda _{j}$ appearing in the pairs $(\lambda _{j} ,1/\lambda _{j})$ are the “squeezing parameters” familiar from quantum optics [1]. When they are all equal to one we have $P=U^{T}\Delta U=I$ and hence $S=R\in U(n)$.

### Multipartite Gaussian states

Multipartite quantum states are usually more difficult to deal with (see [20], Adesso et al. [3] for discussions). Theorem 4 can actually be extended without great difficulty to multi-partite states by iteration. A state $\widehat{\rho }$ is consisting of parts $A_{1},A_{2},\ldots ,A_{m}$ (a “*m* -partition”) with $\dim A_{k}=n_{k}$ is called “separable” if it can be written as a convex sum

33 $$\begin{aligned} \widehat{\rho }=\sum _{j}\lambda _{j}\widehat{\rho }_{j}^{1}\otimes \widehat{\rho }_{j}^{2}\otimes \cdots \otimes \widehat{\rho }_{j}^{m} \end{aligned}$$

where $\widehat{\rho }_{j}^{k}$ is a density operator on $L^{2}(\mathbb {R} ^{n_{k}})$, $1\le k\le m$, $n_{1}+n_{2}+\cdots +n_{m}=m$. It is convenient in this case to adapt the ordering of the phase space coordinates by using the splitting

$$\begin{aligned} \mathbb {R}^{2n}=\mathbb {R}^{2n_{1}}\oplus \mathbb {R}^{2n_{2}}\oplus \cdots \oplus \mathbb {R}^{2n_{m}} \end{aligned}$$

and writing $z=(z^{1}\ldots ,z^{m})$ with $z^{j}=(x_{1}^{j},p_{1}^{j} ,\ldots ,x_{n_{j}}^{j},p_{n_{j}}^{j})$ for $1\le j\le m$.

Theorem 4 extends to the multipartite case:

#### Corollary 6

Every multipartite Gaussian state $\widehat{\rho }$ can be disentangled by a symplectic rotation: there exists $U\in U(n)$ such that

$$\begin{aligned} \widehat{U}\widehat{\rho }\widehat{U}^{-1}=\sum _{j}\lambda _{j}\widehat{\rho }_{j}^{A_{1}}\otimes \widehat{\rho }_{j}^{A_{2}}\otimes \cdots \otimes \widehat{\rho }_{j}^{A_{m}} \end{aligned}$$

with $$\lambda _{j}>0$$ and $$\sum _{j}\lambda _{j}=1$$.

#### Proof

We begin by noting that the conditions (8)–(10) on the covariance matrices extend by induction on *m* to the multipartite case: the Gaussian state $\widehat{\rho }$ is separable if and only if there exist covariance matrices $\Sigma _{A_{j}}$ such that

34 $$\begin{aligned} \Sigma _{A_{j}}+\frac{i\hbar }{2}J_{A_{j}}\ge 0\text { }(1\le j\le m)\text { , \ }\Sigma \ge \bigoplus _{j=1}^{m}\Sigma _{A_{j}} \end{aligned}$$

where $J_{A_{j}}$ is the standard symplectic matrix on $\mathbb {R}^{2n_{j}}$. One thereafter proceeds as in the proof of Theorem 4 by partitioning the eigenvalue matrixin submatrices $\Delta _{A_{j}}$ corresponding to the parts $A_{1} ,A_{2},\ldots ,A_{m}$, that is

35 $$\begin{aligned} \Delta =\Delta _{A_{1}}\oplus \Delta _{A_{2}}\oplus \cdots \oplus \Delta _{A_{m}}. \end{aligned}$$

Setting $\Sigma _{A_{j}}=\frac{\hbar }{2}\Delta _{A_{j}}^{2}$ for $j=1,2,\ldots ,m$ one then verifies, using again (24), that the conditions (34) are satisfied. $\square$
